# Summary findings from a mixed methods study on identifying and responding to maternal and newborn illness in seven countries: implications for programs

**DOI:** 10.1186/s41043-017-0126-9

**Published:** 2017-12-21

**Authors:** Danielle Charlet, Allisyn C. Moran, Supriya Madhavan

**Affiliations:** 1 University Research Co., LLC, Bethesda, MD USA; 2 Bureau for Global Health, United States Agency for International Development, Washington, DC USA

**Keywords:** Maternal mortality, Newborn mortality, Developing country, Qualitative research, Care-seeking behavior

## Abstract

**Background:**

There is a lack of systematic information documenting recognition of potentially life-threatening complications and decisions to seek care, as well as reaching care and the specific steps in that process. In response to this gap in knowledge, a multi-country mixed methods study was conducted to illuminate the dynamics driving Delays 1 and 2 across seven countries for maternal and newborn illness and death.

**Methods:**

A common protocol and tools were developed, adapted by each of seven study teams depending on their local context (Ethiopia, India, Indonesia, Nigeria, Tanzania, Uganda, and Nepal). Maternal and newborn illness, and maternal and newborn death cases were included. Trained interviewers conducted event narratives to elicit and document a detailed sequence of actions, from onset of symptoms to the resolution of the problem. Event timelines were constructed, and in-depth interviews with key informants in the community were conducted. Transcripts were coded and analyzed for common themes corresponding to the three main domains of recognition, decision-making, and care-seeking.

**Results:**

Maternal symptom recognition and decision-making to seek care is faster than for newborns. Perceived cause of the illness (supernatural vs. biological) influences the type of care sought (spiritual/traditional vs. formal sector, skilled). Mothers, fathers, and other relatives tend to be the decision-makers for newborns while husbands and elder females make decisions for maternal cases. Cultural norms such as confinement periods and perceptions of newborn vulnerability result in care being brought in to the home. Perceived and actual poor quality of care was repeatedly experienced by families seeking care.

**Conclusion:**

The findings link to three action points: (1) messaging around newborn illness needs to reinforce a sense of urgency and the need for skilled care regardless of perceived cause; (2) targeted awareness building around specific maternal danger signs that are not currently recognized and where quality care is available is needed; and (3) designing appropriate contextualized messages.

This research links to and supports a number of current global initiatives such as Ending Preventable Maternal Mortality, the Every Newborn Action Plan, the WHO Quality of Care framework, and the WHO guidelines on simplified management of newborn sepsis at the community level. This type of research is invaluable for designing programs to improve maternal and newborn survival to achieve ambitious global targets.

## Background

Improving maternal and newborn survival are central to the global health development agenda, as articulated in the Sustainable Development Goals (SDGs) [1] and Global Strategy for Women’s, Children’s, and Adolescent’s Health, 2016–2030 [2]. To achieve ambitious targets for reducing preventable maternal and newborn mortality, women, families, and care givers must utilize quality maternity and newborn care in a timely fashion. There are a variety of barriers and facilitators to identification of potentially life-threatening illness and timely decision-making and action, especially around the time of birth.

The Three Delays model, developed by Thaddeus and Maine in 1994, has been widely used and adapted by maternal and newborn programs to describe the barriers faced by women and families in accessing and receiving quality care for maternal and newborn complications [3, 4]. The Three Delays include (1) delay in decision to seek care, (2) delay in reaching appropriate care, and (3) delay in receiving care. The MotherCare project adapted the Three Delays model by splitting the first delay into steps: (1) recognition of the problems and (2) decision-making [5]. The importance of understanding household, community, and health system-level factors, and the interactions therein, influencing each point along the three-delay pathway cannot be overstated. Interventions to address these factors, if known, can go a long way in reducing the delays at each stage of the continuum, ultimately improving maternal and newborn survival. Another similar conceptual model that has been used for childhood illness is the Pathway to Survival, which indicates three steps along a continuum to avoiding mortality, including (1) recognition of medical problem, (2) timeliness to decisions and actions and access to care/logistics of referral, and (3) quality of medical care [6].

While there is evidence of a variety of factors that inhibit and facilitate each of the Three Delays as well as the Pathway to Survival, the majority of studies has focused on specific geographic or cultural environments [3]. There is a lack of systematic qualitative information documenting the first two delays around recognition/identification of signs and symptoms of potentially life-threatening illness and decisions to seek care (Delay 1), as well as reaching care and the specific steps in that process (Delay 2). In response to this gap in knowledge, a multi-country set of case studies was implemented by the USAID-funded Translating Research into Action Project (TRAction) to illuminate primarily the dynamics driving Delays 1 and 2 across six countries for maternal and newborn illness and death. An additional study, also USAID-funded, using the same methods and tools was conducted in Nepal and is included in this analysis.

Each of the seven studies provides in-depth examination around processes of illness identification, decision-making, and care-seeking that highlight the importance of understanding local circumstances, beliefs, and practices. Taken together, the findings—in Ethiopia, India, Indonesia, Nepal, Nigeria, Tanzania (findings not presented in this supplement), and Uganda—reveal commonalities between seemingly disparate country settings and articulate the uniformity of differences between cases of maternal and newborn illness. This paper presents a synthesis of findings from across the seven country studies, with a view to reaffirm and challenge assumptions around the drivers of the first two delays. We then discuss implications for policy makers and program implementers who work at the household, community, and/or health system levels.

## Methods

**Fig. 1:**
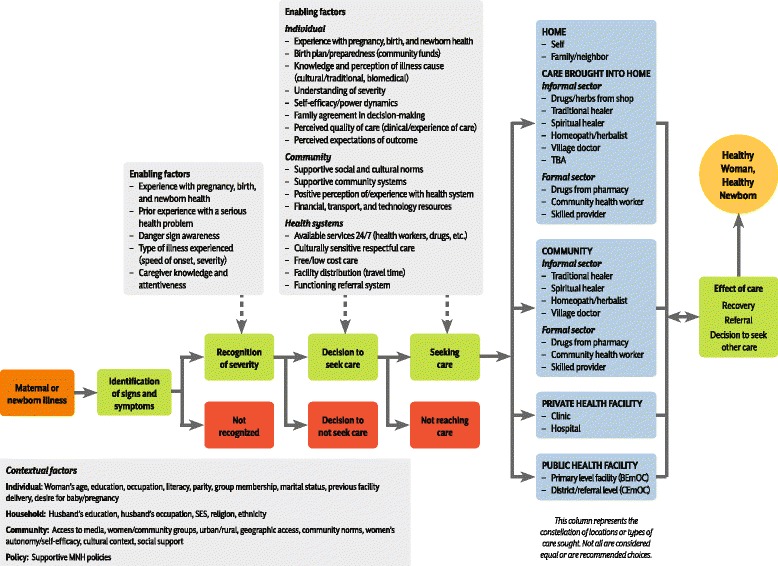
Conceptual framework for recognition and care-seeking for maternal and newborn illness

The study used the Three Delays Model and elements of the Pathway to Survival as the overall conceptual framework. During the study process, the framework was adapted and refined to reflect preliminary findings. The first delay was split into three distinct categories: (1) recognition/identification of potentially life-threatening maternal or newborn illness, (2) recognition/understanding of illness severity, and (3) decision-making process around care-seeking. The second delay focused on the steps of the care-seeking process, including the timeliness of each action (Fig. 1).

Based on an initial consultation in 2014, a common protocol and tools were developed. Each study team adapted the protocol, depending on their local context. Four case types were included: (1) maternal illness (focus on perceived postpartum hemorrhage); (2) newborn illness (any illness); (3) maternal death (any cause); and (4) newborn death (any cause). The inclusion and exclusion criteria were defined by case type, and the same criteria and screening tools were used in all study sites. Case identification varied slightly between study sites; approaches included reports from community health workers, community-based informants, facility-based records, discussions with other health providers, and a household survey (India only). Trained interviewers conducted event narratives with a group of respondents, interviewed together, who were present at the time of the illness to elicit and document a detailed sequence of actions, from onset of symptoms to the resolution of the problem. An event timeline was also constructed for each case, plotting timing of key symptoms, decisions, and actions provided in the interviews. As most of the studies were embedded within or associated with an ongoing program, additional quantitative data were also used to triangulate with the event narrative findings. In-depth interviews (IDIs) and focus group discussions (FDGs) with key informants, such as community health workers, women’s groups, or village leaders, were also undertaken to understand community dynamics, relevant sociocultural beliefs and practices, and health systems issues depending on the study context. Brief facility assessments and community mapping were also carried out to provide additional contextual information. The event narratives, in-depth interviews, and focus group discussions were coded and analyzed for common themes corresponding to the three main domains of recognition, decision-making, and care-seeking. Common tools were developed by TRAction (e.g., questionnaires, code book) and used by all teams. TRAction facilitated trainings of data collectors and data analysis meetings with each study team to ensure consistency of approach across sites, as well as a joint analysis meeting with all study teams to review site-level analysis and discuss emerging cross-site findings. The total sample included 84 maternal illness cases (perceived postpartum hemorrhage), 45 maternal deaths, 83 newborn illness cases, 55 newborn deaths, 64 IDIs/FGDs, and 99 health facility assessments across all sites. A more detailed description of the methods and data related to the common protocol used and specific country studies can be found in a separate paper [7] and in each country paper.

Find a description of study sites in Table 1.

**Table 1:** Study sites by country

Country	Study sites	Population	Total event narratives	Additional IDI/FGDs
Ethiopia	Amhara and Oromiya regions; 6 districts	350,000	51	0
India	Uttar Pradesh state; Amethi and Raibereli districts	2,000,000	32	20
Indonesia	Papua Province, Jayawijaya District	431,338	16	5
Nepal	Sarlahi District	750,000	32	10
Nigeria	Jigawa state; 96 communities	290,000	40	18
Tanzania	Mtwara Region; Tandahimba and Newala districts	433,006	48	5
Uganda	Busoga Region; Mayuge and Namaingo districts	693,000	48	6

## Results

### Identification of signs/symptoms and recognition of severity

The event narratives elicited a range of signs and symptoms identified by individuals, families, and care-givers.

For maternal illness cases, the study focused on signs and symptoms of perceived postpartum hemorrhage (PPH). In general, respondents were able to report signs of perceived postpartum hemorrhage and differentiate between perceived “normal” postpartum bleeding and when the bleeding was indicative of a problem. This was less common in the Nepal study site where recognition of excessive bleeding was generally not reported as prompt. Other maternal signs and symptoms mentioned in the maternal death cases were not always recognized as quickly. For example, headache, swelling, and vomiting were reported in some study sites as normal in pregnancy—in Nigeria, headache was reported as a sign of impending delivery. Convulsions or loss of consciousness were universally recognized as severe.

Across study sites and respondents, a variety of factors indicated a severe or very serious maternal illness. Some respondents reported linking severity with the presence of specific symptoms or clusters of symptoms, while others noted a change from normal behavior, duration/frequency/quantity, and/or recurrence. For example, reported postpartum bleeding was perceived as excessive most frequently by quantity, described as having to change cloths many times, soaking through multiple pads, pooling on a mattress or floor, or filling a container of a certain volume (by demonstrating the volume, for example, showing a plastic bag).

“I was bleeding a lot. It was as a full amount of plastic bag here. Similar to this plastic bag...One plastic bag of blood, and then I was unconscious. It was three times.” (focal woman, Indonesia)

Although respondents acknowledged that some postpartum bleeding was normal, bleeding perceived to be excessive was generally recognized quickly and perceived as dangerous across all study sites, again with the exception of Nepal. In the Nepal study site, even perceived excessive bleeding was not always deemed as dangerous, as described by a traditional birth attendant.

“If the person had fainted or if the person felt nauseous after excessive bleeding then we would consider it as a problem.” (Traditional birth attendant, Nepal)

Identification of signs of newborn illness was more variable: in some study sites, respondents reported timely recognition of a number of symptoms (India, Tanzania), some mixed (Ethiopia—some symptoms well recognized, others not), and some had overall poor recognition (Uganda, Indonesia).

Similar to the maternal cases, across study sites and respondents, there were a variety of factors that indicated a severe or very serious newborn illness; however, in general, there was a lack of understanding of the susceptibility of newborns to rapidly decline, as the presence of signs was frequently not tied to perceived risk or urgency in action. In the majority of newborn cases, both death and illness cases, there was a delay between recognizing signs and seeking care.

“The illness started with coughing and spilling of the milk that he sucked from the breast…the fever became severe and it has got very serious at night that he was coughing for long… the fifth day morning [after the first symptom was recognized] he failed to suck the breast and became so weak…it took us about five days to make a decision that the child needs care, but after we made a decision it was only an hour that we took to get the child to the health center.” (mother of newborn, Ethiopia)

Past experience by self or others also played an important role in recognizing severity. Across all study sites, respondents reported quickly identifying symptoms and perceived seriousness of illness as a result of having previously seen or experienced similar symptoms.

“When [the newborns] started crying, I knew that what took the other child had also come for these ones because their crying is different from the normal crying.” (caretaker, Uganda)

Identification of signs and symptoms of maternal and newborn illness was most often by women themselves, close family members, and birth attendants. Although women, self, or others, were more frequently involved in identification of maternal illness, and particularly symptoms of perceived PPH, husbands did play a role, varying by context. For example, in the Indonesia study, some husbands understood the seriousness of PPH and its potential risk of death:

“Facilitator: So you only stayed with Mama inside?

Husband: Yes, usually if the placenta did not come out it would mean death, so I was scared. I was scared that my wife would pass away, so I stayed with Mama inside.” (husband, Indonesia)

In the Nigeria study, although husbands did not play a role in making a judgment about the severity of PPH, they did play a role in recognizing severity of other maternal signs and symptoms, including fever and headache.

Across settings, mothers and female relatives played a key role in recognizing newborn illness.

### Decision-making

Once signs and symptoms of perceived illness were recognized and identified as severe or serious, there are a variety of factors that influence decision-making around care-seeking for both maternal and newborn cases.

The primary decision of whether to seek care or not varied by type of case (maternal or newborn); cultural norms around pregnancy and childbirth; perception of cause and severity of symptoms; and perception of expected outcome. The interplay of these factors also influences the decision of type of care to seek.

The decision to bring care home (either treatments/medications or providers, including spiritual) versus seeking care outside the home was strongly influenced by cultural confinement periods for newborns and mothers, which provided a strong disincentive for household decision-makers to seek care outside of the house. This was seen, for example, in Uttar Pradesh, India, where local customs dictate a 9-day confinement after birth. If the family decided that care was needed during this period, invariably, care was brought in to the home in the form of medicines, community health worker, or other provider. Moreover, because of this confinement, few family members are in contact with the mother and newborn after birth, and hence, fewer people are able to witness illness and share information up through the decision-making chain. Similarly, in Ethiopia, confinement for mothers and babies was based on strongly held local customs intended to protect them from evil supernatural forces and steered decision-making to home-based care and care brought in to the home.

On a related point, the perceived cause of illness influenced decision-making about the type of care sought (traditional vs biomedical, and home vs facility). If illness was perceived to be supernatural in etiology, decisions were nudged towards seeking care from informal/traditional providers (e.g., shaman, traditional birth attendants (TBAs), and clergy). Similarly, illnesses thought to have a biomedical cause elicited decisions to seek care from the formal sector. In all study sites, respondents reported both biomedical and spiritual causes of illness. Biomedical causes were more commonly reported than spiritual causes in maternal cases, whereas spiritual causes were more commonly reported in newborn cases. Although perceived cause influenced the type of care sought, it did not appear to influence the time to decision-making.

Perceived risk or severity of symptoms served to accelerate decision-making around care-seeking for newborns and mothers across all study sites. In Jayawijaya, Indonesia, maternal loss of consciousness triggered decision-making to seek care outside of the home. In Jigawa state, northern Nigeria, convulsions drove decisions to seek formal care. In study sites in Uganda and Tanzania, babies’ difficulty in breathing motivated people to quickly decide to seek care, though the care-seeking process itself was long and drawn out in many cases.

In some cases, although an illness was perceived as severe, it was not necessarily associated with a high risk of negative outcome (such as death) and a decision was made to delay seeking care. Families in these cases reported recognizing the illness and the need to seek care, but waiting to do so until conditions changed (waiting for morning, for example). This suggests a discrepancy between perceived severity and sense of urgency which may be due to difficulties with travel at night, finding transportation, and other security-related issues. This discrepancy was least common in the maternal illness (perceived PPH) cases, as families generally reported knowing that bleeding postpartum could quickly lead to death. The India study reported different findings where less urgency was reported for postpartum bleeding, at least some of which could have been related to differentiating between “normal” and “excessive” postpartum bleeding or secondary PPH (Most cases were thought to be secondary PPH because most deliveries were in facilities and women stayed at the facilities overnight or for a day. However, these cases were not clinically diagnosed).

The issue of perceived outcome expectation combined with the phenomenon of fatalism features prominently in most settings, but it is unclear what the exact relationship is to decision-making. In Ethiopia and perhaps Indonesia study sites, families’ perception that a severely ailing newborn has an increased likelihood of dying may influence the decision against seeking care or at least against going outside the home to seek care. This, combined with long distances to facilities, can provide disincentives to leave the home to seek care. In the Nigeria study site, at least one respondent expressed the sentiment that the decision to take the woman to the hospital would never have been taken had they known she was about to die. In more cases than not, it appears that respondents’ expression of fatalism is more readily seen as a post hoc measure to cope with loss. Perceptions of quality of care, even poor quality of care, did not seem to deter families from the decision to seek care or initiating the care-seeking process. Families demonstrated a desire to proactively address the illness of a family member, even if there was knowledge that facility-based services would be of poor quality. In study sites in Nigeria, Uganda, Tanzania, and Indonesia, despite commonly accepted understandings that facilities lack staff have shortages of drugs, and even incompetent or disrespectful health professionals, families still made the decision to seek care. Where choice existed, families decided to bypass facilities that were perceived to have poor quality of care in favor of seeking care from “high” quality facilities. However, this choice did not exist for all study sites. Respondents also commonly reported that previous positive experiences with particular providers or facilities, by self or others, were facilitating factors in deciding where to seek care (where choice existed). Quality of care, including medical and user experience dimensions of care, was frequently mentioned by respondents as a major consideration in the decision-making process.

The main decision-maker for initiating the care-seeking process varied between maternal and newborn cases and across study sites (Table 2). In all countries, the baby’s mother was generally the primary decision-maker. Older women in the household also appeared to wield significant influence given their past experience as well as the perception that delivery-related complications are considered a “women’s domain.” In general, regardless of their role in decision-making, the husbands/fathers played a key role in facilitating care-seeking once a decision was made to seek care. The role of women in decision-making about seeking care for PPH, however, was less central. Women were often involved to some extent in such decision-making, but were rarely key decision-makers. Although this could be related to a woman’s condition at the time of decision-making, this did not appear to be the driving reason for their exclusion from the decision-making process.

**Table 2:** Key decision-makers, by country and case type

	Maternal cases	Newborn cases
Ethiopia	Husband, focal woman	Mother and father, broader group of relatives participates
India	Mother, mother-in-law	Fathers, elder women, traditional healers
Indonesia	Husband	Mother, father, “maternal uncle” (senior male relative)
Nepal	Mother, mother-in-law, husband	Entire family, female relatives
Nigeria	Husband, co-wife, female relatives	Mother and father
Tanzania	Husband	Mother and father
Uganda	Focal woman, caretaker, husband, mother/mother-in-law	Mother, paternal grandmother

### Care-seeking

Different patterns were seen for care-seeking for perceived maternal and newborn illness and death. There was considerable variation across cases and study sites; however, some commonalities were apparent. In general, it was more common for the first step of care for the newborn cases to be home-based care (either traditional or spiritual), compared to maternal cases in which care from a skilled provider or facility-based care was the most common first step. Additionally, doing nothing or providing home-based traditional or spiritual care was reported more frequently for newborn than maternal cases.

This finding was related to several contributing factors. The perceived cause of illness tended more towards spiritual causes for newborn illnesses, which directed care-seeking towards traditional or spiritual remedies. In many cases, a spiritual healer was called in to provide counsel and remedies even while preparations are being made to seek formal care. In the Nigeria study, it was common for a spiritual treatment to be pursued prior to seeking medical care; however, there were also examples of spiritual leaders encouraging care-seeking:

“When we saw that the problem has become very serious, we called one Mallam and he supplicated for her, he then asked us to take her to the hospital, because her body has become very cold, he asked us to hurry up and take her, a car was even sent…” (Nigeria)

In this narrative, care was sought sequentially; however, this provides an example of how care-seeking from both traditional/spiritual and formal sources could happen concurrently, which can potentially reduce delays in timely receipt of skilled care.

Related to this, newborns were often perceived as being susceptible to spiritual harm (description of “spiritual harm” varied by country) if taken out of the house too soon after birth, and therefore, home-based treatments were pursued. This, however, does not provide an explanation for why medication or skilled providers were not brought into the home, as was commonly done for maternal cases, particularly in the Indonesia and India study sites (though more community-based providers are present in these settings). Another related factor is the perception that nothing can be done to treat newborn illness or that newborns cannot be saved. As with decision-making, this emerged often in relation to other barriers to care-seeking (lack of finances for services or transportation) and spiritual beliefs (God’s will), which suggests these may be retrospective justifications for decisions made, rather than factors that actively influenced care-seeking.

In some sites, care, or at least counsel, was more likely to be sought from a trusted community health worker for maternal complications than for newborn dangers signs. In the Indonesia site, community midwives were consulted and in the India site, ASHAs (Accredited Social Health Activists) were called. However, these resource persons were rarely sought out for newborn illness.

Another pattern that was apparent in some countries, particularly in the Tanzania and Indonesia study sites, was the bypassing of primary care level facilities. In both countries, and to some extent in others, care was sought from a variety of sources, including care brought home (medications or skilled care), dispensaries, and hospitals, but generally the primary care level facilities were bypassed. It was commonly perceived that adequate services would not be available at these facilities, and this drove families to seek care at other higher level facilities. The opposite was seen in other study sites, like Nigeria, where primary-level facilities were often the first place for care-seeking. Lack of providers, medications, and supplies resulted in delays in receiving appropriate care in several study sites.

**Fig. 2:**
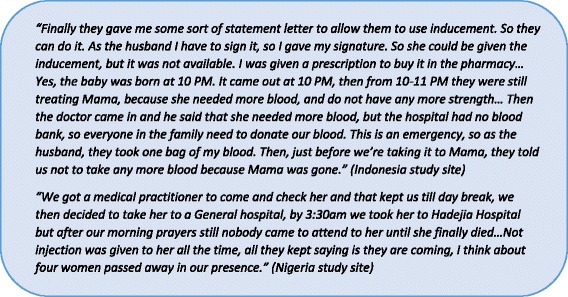
Unavailability of services at facilities

Demand and attempted utilization of health services featured prominently across all settings. Some respondents reported going to three facilities before the illness was resolved or the patient died. Although the study was not specifically designed to investigate issues related to the third delay, this proved to be a common issue across all settings. The numerous steps of care-seeking were frequently driven by unavailable or poor-quality services at a given facility, which created delays in receiving appropriate treatment. Figure 2 illustrates an example of this finding.

Table 3 presents a summary of cross-country findings.

**Table 3:** Summary of cross-country findings

Illness recognition and perceptions of severity						
Maternal	Strong recognition of symptoms of excessive bleeding	Families able to assess severity of perceived PPH	Non-bleeding signs/symptoms not as easily recognized and varies by country	Recognition of severity of perceived PPH usually triggers immediate care-seeking, but not necessarily for other illnesses	Illness generally attributed to biomedical causes	Clusters of signs/symptoms may aid in recognition of severity as well as past experience with similar signs/symptoms
Newborn	Variable recognition of signs of newborn illness	Severity more likely to be acknowledged with longer duration, but poor recognition of risk of rapid decline		Recognition of severity of illness does not necessarily precipitate immediate care seeking	Illness often attributed to supernatural causes	
Decision-making						
Maternal	Key decision makers are husband, mother-in-law, elders	Perceived severity speeds up decision making	Perceived outcome, or belief in unfavorable outcome does not discourage decision to seek care	Community programs (e.g., self-help groups, savings groups) can aid in decision-making	Cultural norms of post-partum seclusion fosters decision-making towards home-based care	Perceived cause (biomedical vs supernatural) influences decision making (skilled vs traditional care)
Newborn	Key decision makers are mother, mother-in-law, older woman	Perceived severity does not necessarily result in prompt decision-making	Perceptions that there is nothing to be done does influence decision to seek care			
Care-seeking						
Maternal	Care is sought outside the home except in cases of cultural norms of post-partum seclusion	In most cases, skilled care sought (although traditional/ spiritual care can also be sought)	Several points of care accessed reflecting supply side failures	Perceived and actual poor quality of facility care does not discourage care-seeking	Trusted community based workers are consulted in care-seeking process	
Newborn	Care more likely to be brought into the home as a first step	Non-skilled care often sought, especially if perceived supernatural cause			Community-based workers are generally not viewed as resources for newborn illness	

## Discussion

This multi-country study sought to systematically document recognition of signs and symptoms of maternal/newborn illness, decision-making processes, and care-seeking patterns across different geographic and cultural contexts. Across these diverse settings, although there were important differences, there were also commonalities which can inform actions for programs aiming to improve maternal and newborn survival.

### Newborns

The difficulty in identifying specific newborn illnesses was seen across all country sites as many signs and symptoms are non-specific. Moreover, it is difficult for a lay person to clearly identify specific issues, especially in settings with postpartum confinement of the woman and baby as well as homes with poor-lighting conditions. This echoes other findings in the literature around illness in children under 5 years of age and particularly around the challenge of predicting the severity of febrile illness in neonates [8–10]. Although mothers and other family members tended to have a good general sense of abnormal behavior for newborns—crying too much, not feeding, vomiting frequently, etc.—they did not necessarily associate the abnormal behavior with risk or sense of urgency. In addition, in most cases, the perceived cause was associated with spiritual factors, which resulted in care-seeking from traditional providers, ultimately delaying skilled care.

This constellation of issues suggests that in many settings, it will be appropriate that messaging for newborns focus primarily on early and immediate care-seeking, rather than differentiating between mild and severe illness. A clear message should be promoted on how rapidly health can deteriorate for a new baby regardless of the cause. Simplified guidance should be given to seek skilled care immediately if any sign of illness is observed regardless of perceived cause, assuming an understanding of the cultural perceptions of “normal” and with contextualized counseling on harmful practices around normal and abnormal symptoms. For example, in the Ethiopia study, a swollen uvula was perceived to be a common cause of newborn illness and removal of the uvula a common practice. Also, the practice of bringing care into the home for newborns across several of the sites indicates a need to reinforce the message of seeking skilled care—whether the provider is brought to the home or accessed at a facility. Although postnatal programs were generally not discussed by respondents, these present a valuable opportunity for identifying illness early if they are implemented in a way that ensure early visits after birth.

### Maternal

Most women and families could identify signs and symptoms of illness (especially for perceived PPH) and understood they were serious and potentially life-threatening, and sought care from skilled health providers. Yet in some settings, there was limited knowledge around specific symptoms of illness other than bleeding, indicating that messages should be targeted to improving recognition of those symptoms that are misunderstood, such as headaches in Jigawa, Nigeria.

“the headache was not that serious, since she was talking and drinking water with her family” (Nigeria)

Although the apparent rapid recognition, decision-making, and care-seeking in the maternal illness cases may be at least partly due to the often obvious and striking nature of PPH, as noted in several of the maternal illness narratives, there were clearly other elements influencing the urgency with which decision-making and care-seeking occurred.

#### Overall

##### Influencing decision-making

While obvious contextual differences exist across country settings, similarities in decision-making are interesting and merit discussion. In general, husbands and elder female relatives played a key role in decision-making for maternal complications, and women themselves often played a limited role, although recognizing that in some cases they may have been physically unable to do so. This is similar to other reports in the literature [11–13]. Positive influencers around decision-making can include self-help groups (SHGs), as seen in the India study site, where women in SHGs were more likely to go to a government facility than to bring care in to the home or access traditional healers, which was seen as a first step of care in the non-SHG households. Similarly, community savings groups, which were reported to facilitate care-seeking in the Uganda study, can serve as a useful example of how financial barriers can be reduced in order to aid in decision-making. Moreover, community health cadres who are viewed more often as maternal health resource persons may also provide an opportunity to be strengthened around newborn care. Community mobilization efforts through participatory learning and action, such as these examples, are recommended in the “Every Newborn Action Plan” [14] and the Ending Preventing Maternal Mortality Strategies document [15] to improve maternal and newborn health, as well as in WHO recommendations [16].

##### Understanding trade-offs in decision-making

While this set of studies has shed light on the decision-making processes around maternal and newborn care-seeking, more innovative methods to tease out this information to inform programming for the poor are needed. Kruk et al. [17] make a strong case for employing behavioral economics to elicit some of the less tangible but critical psychological and behavioral aspects with respect to presentation of choices for health care; providing usable information; and incentivizing care-seeking. For example, drawing from the Nigeria case of a *mallam* advising a family to seek skilled care after performing a spiritual ritual, the scenario could be leveraged to sequence the formal and spiritual care-seeking alongside one another to avoid losing time, while respecting the family’s need to access traditional (non-harmful) measures. The family is more likely to equally prioritize the decision and actions (including the financial implications) to seeking formal care with seeking spiritual care if counseled to do so by a respected *mallam* and if allowed to pursue the ritual as well.

##### Care-seeking and quality of care

The perceived cause of illness was a main driver for type of care-seeking. Spiritual causes were cited across sites, and these perceived causes often drove decision-making processes towards non-clinical care, often resulting in additional delays. Unfortunately, we were unable to explore the different perceived causes in-depth and recommend this area is further explored.

Given the relatively strong and swift illness recognition and decision-making for maternal illness (i.e., perceived PPH) cases, another main barrier was found to be more on the Third Delay in terms of receiving quality care on arrival at the health facility. Across study sites and across maternal and newborn cases, families experienced lack of skilled providers, equipment, and supplies, as well as long wait times exacerbated by needing to find transport and/or funding for transport, services, and supplies/medications. The World Health Organization and UNICEF have recently launched a Network for Improving Quality of Care for Maternal, Newborn, and Child Health including standards for improving quality of maternal and newborn care in health facilities. Between 2017 and 2019, nine countries will join the network including Ethiopia, Nigeria, Tanzania, and Uganda. (http://www.who.int/maternal_child_adolescent/topics/quality-of-care/network/en/).

Several of the study sites reported the phenomenon of by-passing facilities thought to have inadequate care; or bouncing from one facility to another, clearly resulting in delays in securing life-saving treatment. “By-passing” is also reinforced in the literature, indicating that families do weigh the perceived and/or real quality of care in their care-seeking decisions as influenced by prior experience, educational level, and/or type of illness [17–20]. This willingness to bypass or experience with bypassing facilities may actually provide an opportunity to raise awareness in communities as to where adequate services are known to be available for emergencies, with a view to cutting out all other intermediate care-seeking steps. This may also help families to negotiate the difficult choice between trying to access immediate care closer to home and quality care further away.

A related point concerns the demand side. Across all study sites, there was evidence of people trying to seek care even when they did not trust that quality care would be available at the facility level. There is now a need to prevent a decline in demand for services as a result of poor quality of care while supply-side problems, which are inherently longer term systemic issues, are being addressed. As it may be considered “unethical to encourage women to give birth in places with low facility capacity....”, as discussed by Campbell et al. [21], efforts are needed to assess quality of care; map facilities that can provide high quality care, including availability of basic and emergency obstetric and newborn care facilities, and appropriate and timely referrals; and ensure that this information is broadly available in communities.

From these commonalities, specific program recommendations may be as follows:

- *Newborn*: Messaging around newborn illness needs to reinforce a sense of urgency for seeking skilled care, rather than differentiating mild versus severe illness, as newborns’ health can quickly deteriorate.
- *Maternal*: Given the high level of identification of signs and symptoms of perceived postpartum hemorrhage, more targeted awareness-building around specific danger signs that are not currently recognized (depending on the local setting) is needed. In addition, specific messages around where quality care is available should be reinforced to reduce delays in receiving definitive life-saving care.
- *Overall*: Given the varying types of decision-makers for maternal and newborn illness across study sites, it is important to examine each context to identify decision-makers and design appropriate contextualized messages. Also, methods should be employed to understand decision-making, particularly related to what trade-offs families make in decision-making and care-seeking, and the best knowledge available to households. And, messages for illness need to be refined to more clearly indicate where families should go, where quality care is available.
- *Overall*: The findings of this study suggest high levels of care-seeking, especially for maternal complications. The findings also suggest, however, that there are significant gaps around provision of quality maternal and newborn services and there is an urgent need to improve quality of care at facility level.

### Limitations and strengths

This study faced some limitations in methodology and approach which should be taken into consideration when reviewing the results and conclusions. The lack of data around the medical cause of complication and/or death made it impossible to link understanding of symptoms to appropriateness of actions and treatments. However, the main focus of the study was to understand how women and families identified illness based on their perceptions, patterns of illness recognition, and care-seeking irrespective of etiology of illness. Also, because we purposively selected PPH cases, we are able to draw stronger conclusions around the response to perceived abnormal bleeding as opposed to most other maternal and newborn ailments. Although recall was limited to 6 months, there were still recall issues. For example, it was not possible to draw conclusions about timing of specific events and actions. Finally, the low numbers of deaths—especially maternal—relative to illnesses did not allow for a full exploration of the differences in patterns of illness recognition and care-seeking between cases that resulted in death versus cases that survived. Further methods-specific limitations are outlined in Moran AC et al. [7].

Still, the main strength of using a common protocol across seven countries is the ability to pool in-depth qualitative data around especially mortality which would otherwise be very time-intensive to secure. Despite the diversity of settings across countries and continents, the pooling allows for common threads and themes to surface, making these observations more robust. The event narrative methodology also allows for disagreements to be discussed and resolved to obtain consensus on the details of how and why events transpired—something that would not be possible with individual interviews. This was generally positive and resulted in rich findings. There were some disadvantages around getting the group together for the interview as well as preference in some sites for female only groups. These issues were addressed through conducting separate interviews with men. Finally, the focus of the event narrative methodology was squarely on the how and why and less so on the when, what, where, and who. This aspect of the study has contributed to eliciting richness in detail that many previous studies have not been able to do to advance our understanding of why families and caregivers take the actions that they do around maternal and newborn illness. For example, in household surveys, it is possible to ask about the details of care-seeking events, but it is not possible to capture the detail around decision-making processes or to elicit different opinions.

## Conclusion

The importance of this type of research to further understand the barriers and facilitators to appropriate and timely care-seeking cannot be overstated. A major value of this work is that it has drawn out common findings across seven countries from which general recommendations can be built. The implementers and researchers involved in conducting these studies have concluded that all programs should include formative research where information is gathered to better understand the issues within each cultural context and to refine approaches and messages. An example of when this could prove valuable will be as countries start testing approaches to implement the WHO guidelines for community management of possible serious bacterial infection (PSBI) in young infants [22]. In Ethiopia, for example, while training and supervision of frontline health workers on PSBI has improved service accessibility, families are not bringing their sick newborns in to the facilities to receive care—indicating the need to elicit qualitative information as to why this is the case. Given the importance of household- and community-level identification and management of care, understanding the context in which the guidelines are being implemented will be crucial to successful tailoring of messages and implementation.

Advocacy at the global level mirrors this sentiment. A call to action in the recent Maternal Health Lancet Series underscores the need for a prioritization of context-appropriate adaptation of recommendations and implementation strategies around global recommendations for the content of care. Similarly, the Every Newborn Action Plan [14] calls for “each government to conduct a systematic situational analysis and agree to a set of core interventions and packages that match the local context, are relevant to the burden of neonatal morbidity and mortality, and fit within the continuum of care.” In addition, the Core Group has recently launched a Call to Action titled “Complexity Matters” [23] which essentially, promotes the alignment of global efforts around monitoring and evaluation of social and behavior change. Specifically, this Call to Action champions “the use of participatory, narrative, mixed methods and learning based approaches that align with what we know about the context and complexity of SBC program implementation.” This study demonstrates the value of understanding context and behavior to inform meaningful interventions to promote maternal and newborn survival.

## References

[1] UNDP. Sustainable Development Goals. http://www.undp.org/content/undp/en/home/sdgoverview/post-2015-development-agenda.html. Accessed 13 Dec 2016.

[2] Every Woman Every Child (2015). Global Strategy for Women’s, Children’s and Adolescents’ Health.

[3] Thaddeus S, Maine D (1994). Too far to walk: maternal mortality in context. Soc Sci Med.

[4] Wasiwa P, Kallander K, Peterson S, Tomson G, Pariyo GW (2010). Using the three delays model to understand why newborn babies die in eastern Uganda. Tropical Med Int Health.

[5] Koblinsky M. On the pathway to maternal health—results from Indonesia. MotherCare Matters. 1995;5(1):1–2.

[6] Waldman R, Campbel CC, Steketee RW (1996). Overcoming remaining barriers: the pathway to survival (current issues in child survival series).

[7] Moran AC, Charlet D, Madhavan S, Aruldas K, Donaldson M, Manzi F, Okuga M, Rosales A, Sharma V, Celone M, Brandes N, Sherry J, Wall S. Methodology for a mixed methods multi-country study to assess recognition of and response to maternal and newborn illness. J Health Popul Nutr. (41043-36-S1-S1 in this issue) 10.1186/s41043-017-0119-8 PMC5764055 29297390

[8] Abdulkadir MB, Abdulkadir ZA. A cross-sectional survey of parental care-seeking behavior for febrile illness among under-five children in Nigeria. Alex J Med. 2016; 10.1016/j.ajme.2016.02.005

[9] Kassam R, Collins JB, Liow E, Rasool N (2015). Caregivers’ treatment-seeking behaviors and practices in Uganda—a systematic review (part II). Acta Trop.

[10] Rutebemberwa E, Kallander K, Tomson G, Peterson S, Pariyo G (2009). Determinants of delay in care-seeking for febrile children in eastern Uganda. Tropical Med Int Health.

[11] Somé DT, Sombie I, Meda N. How decision for seeking maternal care is made—a qualitative study in two rural medical districts of Burkina Faso. Reprod Health. 2013; 10.1186/1742-4755-10-8. 10.1186/1742-4755-10-8 PMC3575275 23391047

[12] Essendi H, Mills S, Fotso JC (2011). Barriers to formal emergency obstetric care services’ utilization. J Urban Health.

[13] USAID Maternal and Child Health Integrated Program (2012). Cultural barriers to seeking maternal health care in Ethiopia: a review of the literature.

[14] World Health Organization, UNICEF (2014). Every newborn: an action plan to end preventable deaths.

[15] World Health Organization (2015). Strategies toward ending preventable maternal mortality (EPMM).

[16] World Health Organization (2014). WHO recommendation on community mobilization through facilitated participatory learning and action cycles with women’s groups for maternal and newborn health.

[17] Kruk ME, Hermosilla S, Larson E, Mbaruku GM (2014). Bypassing primary care clinics for childbirth: a cross-sectional study in the Pwani region, United Republic of Tanzania. Bull World Health Organ.

[18] Kanté AM, Exavery A, Phillips JF, Jackson EF (2016). Why women bypass front-line health facility services in pursuit of obstetric care provided elsewhere: a case study in three rural districts of Tanzania. Tropical Med Int Health.

[19] Salazar M, Vora K, De Costa A. Bypassing health facilities for childbirth: a multilevel study in three districts of Gujarat, India. Glob Health Action. 2016;9:32178. doi:10.3402/gha.v9.32178. 10.3402/gha.v9.32178 PMC4992671 27545454

[20] Kahabuka C, Kvåle G, Moland KM, Hinderaker SG. Why caretakers bypass primary health care facilities for child care-a case from rural Tanzania. BMC Health Serv Res. 2011; 10.1186/1472-6963-11-315. 10.1186/1472-6963-11-315 PMC3234197 22094076

[21] Campbell OM, Calvert C, Testa A, Strehlow M, Benova L, Keyes E, Donnay F, Macleod D, Gabrysch S, Rong L, Ronsmans C, Sadruddin S, Koblinsky M, Bailey P (2016). The scale, scope, coverage, and capability of childbirth care. Lancet.

[22] World Health Organization (2015). Managing possible serious bacterial infection in young infants when referral is not feasible.

[23] Coregroup. A call to action. Complexity matters: Aligning the Monitoring and Evaluation of Social and Behavior Change with the Realities of Implementation. 2016. https://coregroup.secure.nonprofitsoapbox.com/storage/documents/Social_Behavior_Change/calltoaction.pdf. Accessed 8 Nov 2016.

